# Automated monitoring of dairy cow body condition, mobility and weight using a single 3D video capture device

**DOI:** 10.1016/j.compind.2018.02.011

**Published:** 2018-06

**Authors:** M.F. Hansen, M.L. Smith, L.N. Smith, K. Abdul Jabbar, D. Forbes

**Affiliations:** a Centre for Machine Vision, BRL, UWE, Bristol, UK; b Kingshay Farming & Conservation Ltd, Glastonbury, Somerset, UK

**Keywords:** 3D imaging, Body condition score, Lameness, Weight

## Abstract

Here we propose a low-cost automated system for the unobtrusive and continuous welfare monitoring of dairy cattle on the farm. We argue that effective and regular monitoring of multiple condition traits is not currently practicable and go on to propose 3D imaging technology able to acquire differing forms of related animal condition data (body condition, lameness and weight), concurrently using a single device. Results obtained under farm conditions in continuous operation are shown to be comparable or better than manual scoring of the herd. We also consider inherent limitations of using scoring and argue that sensitivity to relative change over successive observations offers greater benefit than the use of what may be considered abstract and arbitrary scoring systems.

## Highlights

• 3D imaging for concurrently monitoring cow body condition, lameness and weight.
• Novel rolling ball software tool is proposed for body condition assessment.
• Original moving spine segmentation/modelling approach in 3D postulated.
• Real-world performance that is comparable or better than manual scoring.
• Limitations of conventional scoring discussed and a learning approach introduced.

## 1 Introduction and context

### 1.1 The need for precision on the farm

Over recent years there has been a growing interest in exploring the benefits of so called ‘smart farming’. Sometimes also known as ‘precision agriculture’, smart farming has its motivational origins in developments first applied in industrial manufacturing and essentially concerns the use of monitoring and intervention techniques, realised in application through the deployment of sensing technology and automation. In more general terms, smart farming may be defined as the application of innovations in engineering and technology to offer positive interventions in farming activities. One such important agri-tech application concerns the management of dairy cattle, where the provision of appropriate and timely data can help reduce waste and environmental pollution as well as improve animal welfare and farm productivity.

### 1.2 More data, more often

Poor body condition or lameness in cattle, where the latter is often evident as reduced mobility, can negatively impact on both individual animals as well as the wider farm operations. It is well known that the early detection of health problems within the herd can offer significant animal benefits [[1], [2]], such as reduced discomfort, pain and an increased lifespan, as well as offering important advantages for the herdsman in the form of increased milk production, and reduced feed and veterinary costs. The use of cattle monitoring techniques is therefore rightly considered to potentially offer major benefits to both the herdsmen and the animals in their care. However, to be an effective management tool the monitoring of the herd’s status and wellbeing must be undertaken regularly, particularly if trends are to be detected and problems identified early enough to allow more effective interventions to be deployed. Here we argue that effective and regular monitoring is not currently practicable, or indeed taking place [3], and that a new approach is therefore needed. To address this requirement we propose a low-cost automated system for the unobtrusive and continuous monitoring of cows. The technology aims, for the first time, at providing the herdsman with differing forms of related animal condition data acquired simultaneously using a single device. The proposed device will monitor the entire herd daily, affording the necessary and timely information needed to both optimise individual animal nutrition and to identify and prioritise tasks in order to avoid future problems through early and so more effective interventions.

## 2 Simultaneously monitoring of multiple animal condition traits

Measurement of weight, estimation of body condition and checking for lameness are all well-established tools that are commonly used for the purpose of monitoring cattle. Unfortunately, at present these metrics are in general not utilised effectively, as they are most often monitored inaccurately, infrequently and although related, in isolation, i.e. on separate asynchronous occasions using differing techniques or technologies [[4], [5], [6], [7]]. However, such health metrics are by no means independent of each other; rather, they are closely linked. Both Randall et al. and Green et al. [[8], [1]] have shown how cows with poor body condition are at significantly greater risk of developing lameness and that lameness itself links closely with weight loss and other productivity related issues, such as reduced fertility and milk yield.

### 2.1 Manual vs automated monitoring of multiple traits

Conventionally, estimating body condition and detecting lameness are performed by trained observers using established manual scoring techniques. These methods are inherently labour intensive, highly subjective and the results obtained very often inaccurate and inconsistent [[5], [9], [7]]. Humans are also poor at detecting small or subtle change [10], particularly over many observations and extended time periods. However it is precisely these small, relative and gradual differences, rather than absolute spot values, which are most often the more informative measures – for example in the early detection of the onset of lameness. Humans are also generally unable to monitor for more than one trait at a time. For instance, checking for lameness usually necessitates observation from a considerable stand-off distance, needed to view the animal’s overall posture and gait, while assessment of body condition usually requires inspection at close quarters, to assess fat deposits, and may even include palpating the animal. This forces a time, space and correlation separation in manual observation activities, which often leads to irregular, isolated and so uncorrelated evaluations. Furthermore, the presence of human observers may in itself serve to randomly disrupt the very thing they are there to monitor, as for example when cows instinctively alter their behaviour in order to mask their lameness [11]. Animal weight can also be estimated manually (using suitable anatomical measurements), and although automated weighing devices have become relatively more established, the technology remains extremely costly, is also fixed and bulky, and by interfering with normal dairy farm activities may introduce delays and animal stress, thereby altering observed behaviour. For these reasons animal weight may in practice not be regularly captured. Alternatively, combined weight and locomotion traits might be automatically captured and assessed using force plates or in the case of the latter using worn body sensors, however neither of these two technologies can provide a body condition measure. Only a vision based approach offers the potential to combine all three metrics.

## 3 The case for a different approach

Here we first consider further shortcoming in existing manual monitoring techniques, then present a comparative summary of state-of-the-art technologies in the application of automation. The current scoring systems, eg the Pennsylvania State University Scoring Method [4] for body condition and the Sprecher scoring system [12] (or the ‘DairyCo. Mobility Score’ based on [13] – often used in the UK) for lameness, have been developed in the context of, and for, manual use and as they consists of quite generalised qualitative descriptors are arguably not at all best suited to an automated approach or indeed even investigating automated solutions. In the efforts made by the wider research community to date to develop useful monitoring systems, we argue that the use of benchmarking against existing subjective and unreliable manual forms of classification may be less valuable than instead striving for consistency and sensitivity, and evidence thereof, in repeated automated measurements and in the detection of small changes. This is informed by the notion that a relative change in animal metrics over time may be considerably more informative than isolated absolute values. One such example concerns the use of existing lameness classification systems, which fail to model important individual animal idiosyncrasies. This becomes problematic in common cases where healthy animals exhibit what appear as established lameness traits, such as back arching [14], as a legacy from historical injury or simply have a tendency to randomly behave in an unusual and misleading manner. It may therefore be better to establish trends, or in other words what is considered ‘normal’ for each individual healthy animal over many repeated observations, and then to monitor for small change from that norm over time. Only automation can achieve this for the whole herd. Van Nuffel et al. [7] explore the concept of abnormal locomotion due to lameness and go on to review studies on sensor technology to measure changes in gait or behaviour related to lameness. Practical issues are also considered, including trade-offs between high sensitivity and high specificity in the context of false detection and when to treat lameness [7]. It may be the case that given the fundamental desire to use monitoring to improve animal welfare and farm productivity, that automated assessment should abandon any reference to conventional condition scoring systems altogether and instead seek to correlate changes in observational features relating to body metrology, morphology and animal behaviour with readily measured inputs and outputs concerning such things as feed and veterinary costs, milk yield and fertility and with benchmarking such as veterinary examination, level of cytokines in blood samples indicating inflammation etc. – all of which can be easily and objectively quantified.

Many of these considerations draw into question the wisdom of using manual measurements as a base-line or as a ground truth reference for the performance assessment of automated techniques and even the entire proposition of aiming to replicate manual scoring using automation. It is clear however, and arguably now well established, that in all cases there is a need for automation of the monitoring process itself, as existing manual monitoring techniques are frequently unreliable, inconvenient or costly. They can often disrupt the normal farm routine and introduce undesirable animal stress, eg as when palpating a cow in a crush. Most significantly, they are not able to simultaneously monitor for multiple inter-related animal condition characteristics and do not lend themselves to continuous daily data capture, essential to identify evolving trends.

### 3.1 The state-of-the-art in automation

Table 1 provides a summary comparison of some recent approaches to the automated monitoring of cattle reported in the scientific literature as well as several significant patents. It is clear from the published literature to date that no one system delivers all three monitoring functions (for lameness, BCS and weight) from the same compact device – as we will propose here.

**Table 1:** Comparison of cattle monitoring systems described in the academic literature and in patents; NB our proposed system uniquely simultaneously estimates BCS, mobility and weight using 3D data.

Publications	Technique	Lameness	BCS	Weight
[9]	2D		x	
[15]	2D	x		
[16]	2D		x	
[17]	2D		x	
[18]	2D		x	
[19]	Force Plate	x		
[20]	Force Plate			x
[21]	3D		x	
[22]	2D		x	
[23]	2D	x		
[24] & [57]	2D Thermal		x	
[25]	Lying monitors	x		
[26]	3D			x
[27]	3D			x
[58]			x	x
[28]	Force Plate	x		
[29]	Accelerometer	x		
[30]	Force Plate	x		
[31]	2D Thermal			x
[32]	2D	x		
[33]	Force Plate + 2D	x		
[34]	Force Plate + Video	x		
[35]	Force Plate	x		
[36]	Force Plate	x		
[37]	3D		x	
[38]	3D		x	
[39]	Video	x		
[40]	3D			x
[41]	3D		x	
[42]	2D			x
[43]	2D			x
[44]	3D		x	
Patents				
[45]	2D + 3D	x		
[46]	2D + Thermal		x	
[47]	2D			x
[48]	2D		?	x
[49]	3D		x	
[50]	3D		x	
[51]	3D		x	
[52]	Force Plate	x		

## 4 A new approach

Here we will explore a methodology for simultaneously monitoring multiple animal health parameters, specifically body condition, weight and lameness in dairy cattle, using a common dataset, captured twice daily using a single device. This avoids the need to perform separate measurements using different devices. As is the convention, the results obtained will be compared both with established manual assessment (as is normal in the literature) or with calibrated scales in the case of weight, but also given the discussion above, by examination of the raw data outside the manual assessment scoring process.

The rest of this paper is structured as follows. First we introduce the farm-based equipment used for the data capture. Then, after describing the image pre-processing steps, algorithms for weight estimation, body condition and lameness scoring are each introduced. The experimental work is next presented, including results for the three respective condition traits. We finish finally with discussion/conclusions and future work.

### 4.1 Data capture – the equipment

**Fig. 1:**
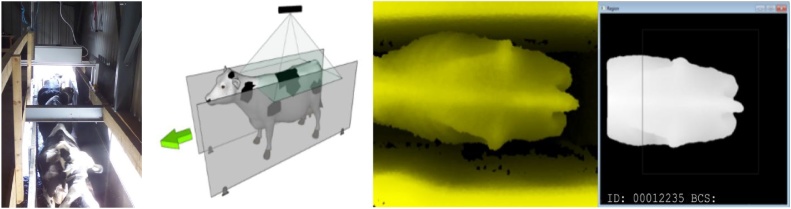
System configuration shown by the two left images. The depth camera is positioned above the cows which pass unconstrained beneath. The narrow race permits only one cow through at a time. A raw depth image of a cow is shown in the middle-right (the camera’s approximate field of view is also shown) and a screen shot of a processed cow is shown on the far-right.

The physical configuration of the data capture system along with example acquired and processed images are shown by Fig. 1. A 3D Kinect-like depth camera views the back of the animal from above as it freely passes along a narrow walkway beneath (two left-hand images). The walkway floor comprised a concrete non-slip surface. The same floor surface was also used during manual observations in order to avoid introducing any change of gait. The cattle, who are accustomed to traveling through the walkway, pass one-by-one in a normal unconstrained manner twice a day after exiting the milking parlour. The 3D camera, which is mounted 2.3 m above the ground to give a 2.5 m horizontal FOV, and computer system are contained in IP66 rated waterproof boxes. A Radio Frequency Identification (RFID) reader, used to identify each cow, is positioned to one side at the cow’s head level, and triggers the image acquisition as the cow approaches. The two right-hand images show the raw 3D image and processed image respectively. The data captured consists of a dense cloud of 3D surface data points, acquired at 30 frames per second.

### 4.2 Data analysis

Once the data are captured, we first undertake data pre-processing and then data analysis in order to extract measures for the traits we wish to monitor.

#### 4.2.1 Image pre-processing

**Fig. 2:**
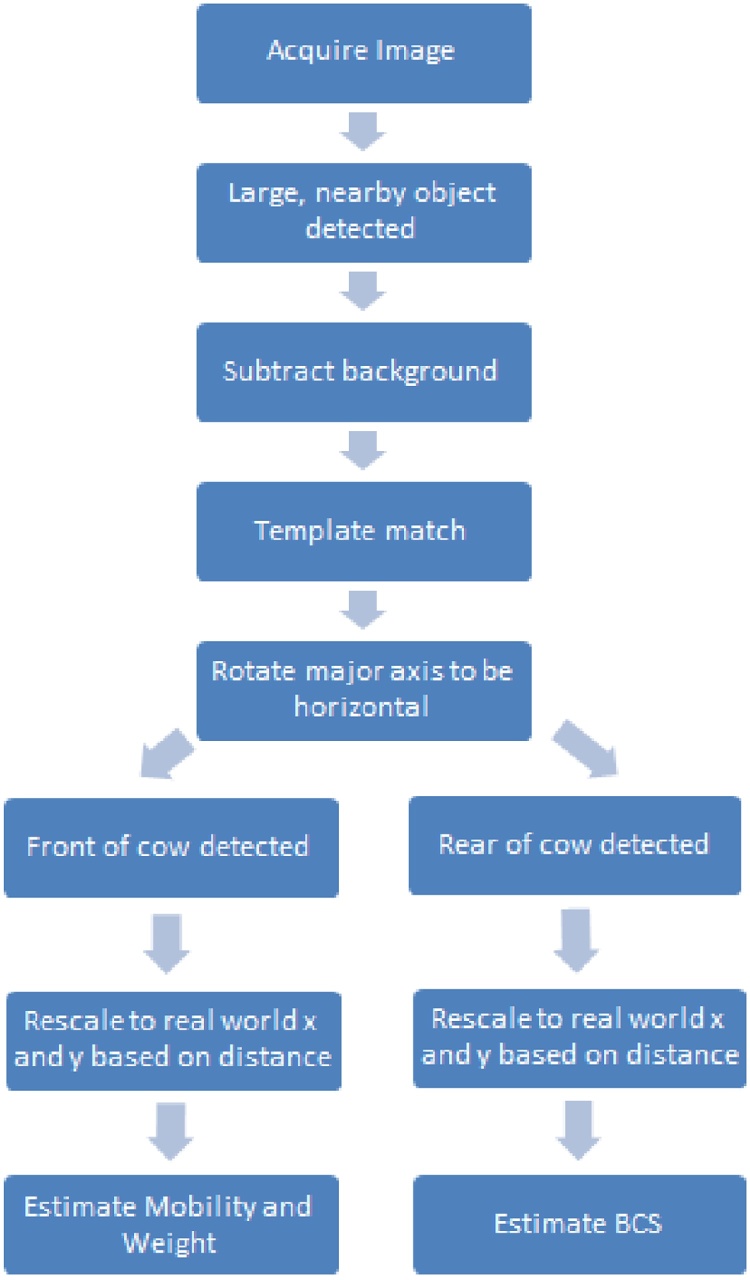
Pipeline of the image pre-processing steps used for automated assessment of body condition (BCS), mobility and weight.

The pre-processing steps (shown in Fig. 2) are required to produce depth data suitable for use by the algorithms which perform the measurement. These major steps in the image pre-processing pipeline are first described, before each of the measurement algorithms for body condition, mobility and weight.

**Fig. 3:**
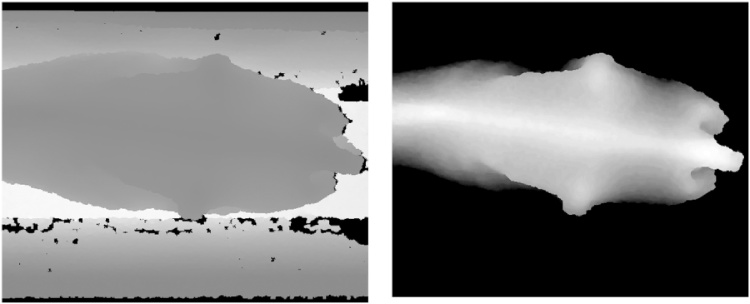
Raw depth image from the 3D camera (left) and the same image with the background removed and thresholding applied (right).

Pre-processing is used to segment the cow (if present) by removing all extraneous information from the scene. The 3D depth camera allows this step to be much more straightforward than would be the case for a standard 2D colour camera. A background image is first taken during set-up when no cows are present. During data capture the image frame is inspected for an object which is closer than 1.5 m, with an area of at least 500 px. Once detected the background depth image is subtracted to leave just the cow visible. This has the effect of removing the side walls and floor. If a cow is present in the frame, then the difference between the background image and the current frame will be significant for those pixels. This is used as a mask through which non-background objects are selected for further processing. A range threshold (1.35 m in this case) is also applied to discard pixels further away, in order to remove parts of the animal which are not needed for further processing (e.g. legs). In order to remove any noise, the largest single area of pixels (the cow) is isolated and all other areas are removed. The effect of this pre-processing can be seen in Fig. 3.

**Fig. 4:**
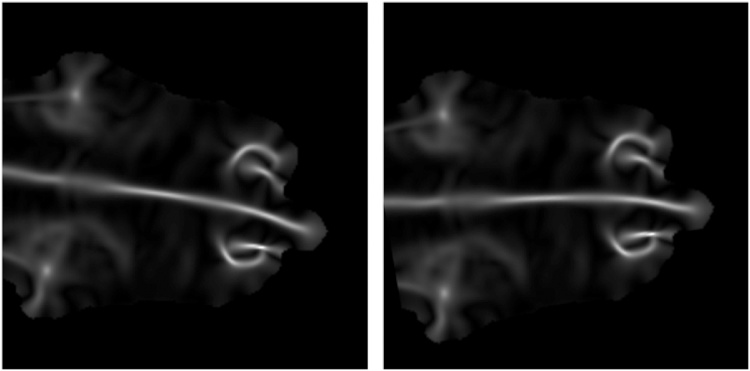
The curvedness data calculated from the depth image of the cow. Before (left) and after (right) rotation has been automatically performed. Notice how the most acutely curved areas show clearly. This is used in template matching for cow region detection, and then in later processing for extracting information about the arch of the spine.

Once all the extraneous information in the frame has been removed, template matching is used to classify whether a front, rear, or front and rear cow region is visible. Given that the matching method needs to be very efficient, a basic windowing procedure is followed using a thresholded normalised cross-correlation matching algorithm, to score how closely a region matches a template. If a region is similar enough to the template then it is used for further processing. It was found that curvedness data rather than the raw depth data (see Fig. 4) offered more robust template matching. Curvedness was first proposed by Koenderink [53] as a measure of 3D shape and is given by $\bar{C}$ in Eq. (1). It is the normalised magnitude of the combined principle curvatures (*κ*_1_, *κ*_2_) corresponding to orthogonal axes, which describe a point on the object's surface, and is calculated from the Gaussian and mean curvatures of the surface.

(1) $$\begin{array}{c} C_{x,y}=\sqrt{\kappa_{1\left(x,y\right)}{}^{2}+\kappa_{2\left(x,y\right)}{}^{2}}\\ C_{max}=\max\left(C\right)\\ {\bar{C}}_{x,y}=\frac{C_{x,y}}{C_{max}} \end{array}$$

In order to align the images for further processing, the found region is rotated so that the major axis sits horizontally in the frame. This is done by thresholding the curvedness data so that only those values in the top 5% remain and extracting the bounding rectangle of the longest region. This then provides the angle by which the cow needs to be rotated in order for that bounding rectangle to lie horizontally across the image frame. An example of the results of this can be seen in Fig. 4.

Finally the x and y coordinates must be rescaled to match the z (depth) scale. The values for the z axis are in millimetres, so we convert the x and y coordinates to also be in millimetres. Given that the distance from the camera to the cow and the lens parameters of the camera are known, it is possible to calculate the real-world dimensions of the FOV (i.e. the width and height in millimetres that each pixel represents). From this the scaling factor for the x and y axis is derived and a transform applied to the data so that the x, y and z axis are all scaled to millimetres. Eq. (2) gives the width and height of the FOV at a given depth.

(2) $$\begin{array}{c} w=2\left(d\times \tan\left(\frac{\theta_{horz}}{2}\right)\right)\\ h=2\left(d\times \tan\left(\frac{\theta_{vert}}{2}\right)\right) \end{array}$$

where w and h are width and height in millimetres across the FOV at distance d from the camera (attained from the depth map). *θ*_*horz*_ = 58° and *θ*_*vert*_ = 45° are the horizontal and vertical angles of the lens. The processed image is next ready to be used to determine physiological parameters relating to the cow as the image now contains segmented data indicating the true dimensions of the surface of the cow: width, height and depth.

#### 4.2.2 Body condition

Body condition scoring (BCS) is a technique used to inform feeding strategy conventionally based on a manual assessment of the energy reserve or nutritional status of the animal. The assessment tends to focus on the level of subcutaneous fat apparent at the rear of the animal around the tail head, hook and pin bones. A fatter cow with a rounded shape is deemed to have a higher score than a leaner cow exhibiting a more angular bony shape, on the typically used manual 1–5 scale (Pennsylvania State University’s Scoring Method). Prior work using both 2D and more recently 3D imaging has sought to find ways of quantify this angularity – see summary in Table 1. Here a 3D assessment technique is introduced that lends itself very well to automating this analysis.

A novel morphological technique for quantifying the angularity of 3D particles was first introduced by Lee et al. in 2007 [54]. The approach extended earlier work [55] aimed at smoothing discontinuities and removing noise from 2D grey level images and is analogous to rolling a ball over the underside of the 3D surface. Where the surface is smooth with relatively low curvature, the ball will make good contact with the surface at every point; however the ball will be unable to make contact with surface points contained within protrusion of high curvature, such as the more angular bony regions of the cow body shape. By quantifying how well a ball of given size fits the 3D surface captured from the animal, a measure of angularity can be obtained and if desired related to the existing manually estimated 5-point classification. This project represents the first time this technique has been applied to estimating body condition. Importantly, the rolling ball algorithm operates globally across the surface and avoids any need to detect and track local features, eg hooks or pins, sometimes required by other methods.

The rolling ball may be simulated by a morphological opening operation (an erosion followed by a dilation) applied directly to the depth image (Eq. (3)).

(3) A ○ B = (A ⊝ B) ⊕ B

A rolling ball with a diameter of 70 px was found experimentally to provide the most discriminating results on a range of cows from BCS 2.25–4. It would also be possible to implement a scheme using different sized balls to see which fits best, or select the ball size as a function of the size of the cow.

#### 4.2.3 Lameness

The detection of lameness, apparent as an alteration in mobility, is based here on the arching of the cow’s spine, where it has been observed that such arching occurs in many (although not all) cases of lameness [56]. When walking, the curvature of the spine can provide an early indicator of discomfort caused by lameness [12]. As such, a cow will show a hunching of the spine at an earlier stage when walking than when stationary. In this system we accurately and reliably extract the spine region as a ‘snake-like’ entity using curvature information and then fit a second order polynomial in order to estimate lameness severity and if desired a correlation with a mobility score. In order to isolate the spine, a ROI is first extracted using a curvedness threshold. Next a polynomial is fit between the highest points in that ROI. Curve fitting can then be performed in both the vertical x-z and horizontal x-y planes to give a 3D snake-like curve that appears to wiggle through space as the animal steps forward. Here therefore lameness severity is related to the magnitude of curvature, while a closer analysis of the curve shape can be used to better localise the particular lame limb. Eq. (4) gives the spine curve polynomial.

(4) $$ax^{2}+bx+c$$

The first coefficient *a* relates to the magnitude of, and direction of, the curve in a vertical plane. A large negative value indicates a more extreme upward facing parabola, whereas a large positive number indicates a more extreme downward facing parabola. The second coefficient *b* effectively gives the skew of the arch, or the degree to which it is shifted right or left. A threshold is set, and if this is exceeded then the cow is identified as being lame. However, because the system continually monitors the cows, it should be possible to build up a model of what happens as a cow gradually becomes more severely lame, and then track this in reverse to be able to predict limits at which a farmer can be informed before more significant lameness occurs.

The system currently only uses the first coefficient, however we are currently investigating the use of the second to provide more accurate information on which limb is affected, as it is hypothesised that this will correlate with the front to back position of the lameness. In future work we will also examine the use of the lateral (ie in a horizontal plane) curvature of the spine in much the same way in order to distinguish between left and right lameness. If the cow is lame on its right-hand side, then the stride length will be shortened on that side. In order to compensate for this, the cow curves its spine in such a way as to minimise the distance between front and back hooves. Thus we expect that such a cow will show a curved spine with the point of the parabola pointing contra-lateral to the affected limb. We should therefore be able to also localise the lameness with regards to the left or right hand side of the cow. It is important to understand there are other factors which can cause curvature changes to the spine not related to mobility, such as craning of the neck. However these types of movement are far more common in a stationary cow rather than a moving cow, thus we ensure that measurements are discarded if the cow is not in motion.

#### 4.2.4 Weight

Our weight estimation is obtained by integrating the pixels of known area over their depth to produce an estimate of volume and assumes the density *D* of water to give an animal mass *M* estimate in kilograms (Eq. (5)).

(5) $$M=\left[\int_{x=0}^{x=N_{x}}\int_{y=0}^{y=N_{y}}h\left(x,y\right)\delta x\delta y\right]\times D$$

The discrete version is given in Eq. (6), where Nx is the number of columns and Ny is the number of rows in the pre-processed image, D is the density of water, and h(x, y) is the height at pixel (x, y).

(6) $$M=\left[\overset{x=N_{x}}{\underset{x=0_{}}{\sum }}\overset{y=N_{y}}{\underset{y=0}{\sum }}h\left(x,y\right)\right]\times D$$

For each image frame in which the front of a cow is detected, an estimate is produced and stored. The largest value for each cow is then used as the final weight estimate. This corresponds to the largest view of the animal, and omits the head and neck region. However, because it is assumed that the volume is a solid body from the surface of the cow's back to the floor (i.e. no legs are modelled), the mass of the omitted regions is compensated for.

The three processes described above have been implemented on the farm within a single device. The system is able to identify a single cow from a herd using its RFID tag.

## 5 Experimental work including results

A herd of around 200 Holstein-Friesian cows were passed through the system. A consensus of averaged manual BCS scores was generated by three trained staff using the 5-point Pennsylvania State University Scoring Method [4]. Manual lameness scoring, again performed by a consensus of trained observers using the 5-point Sprecher scoring system [12], and scale weighing were also undertaken. All observations were performed immediately after milking. Details of the manual scoring process, including observed descriptors, are provided in the references. Logistical problems required that the manual BCS scores had to be taken the following day; however it was considered unlikely the cows would have changed significantly in a single day. In order to test repeatability, 16 cows were selected from the herd and passed under the system up to five times on the same day. All readings were taken in under an hour. Automated results were not generated for all cows, mainly due to either cow aberrant behaviour, eg a cow has her tail out or a following cow obscured the rear of the current cow with her head, or the RFID system did not successfully detect a tag. In all cases the aim was to ensure erroneous data could be automatically detected and removed from the analysis.

### 5.1 Results for body condition assessment (linking angularity with BCS)

**Fig. 5:**
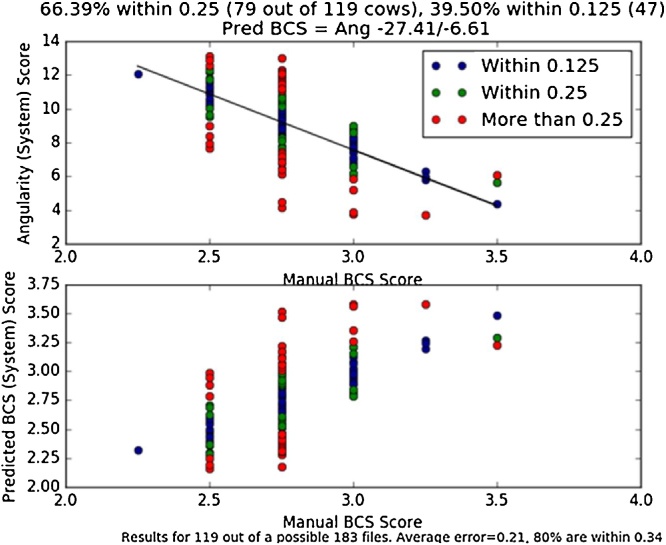
Manual BCS vs the angularity (top) and manual BCS vs the predicted BCS (bottom) for the 199 cows that were automatically scored, where a BCS of 1 = emaciated and a BCS of 5 = very obese.

From ∼200 cows on the farm, 119 were automatically scored. The two graphs in Fig. 5 show the manual score vs the angularity (top) and the predicted BCS (bottom). A clear trend is visible, with blue and green dots indicating a predicted BCS score within an eighth and quarter score of the manual scores respectively. Red dots indicate a result that is more than a quarter score from the manual score. The average error across all 119 cows is only 0.21 (ie less than a quarter score) and 80% of the cows were scored within 0.34 of the manual observation.

**Fig. 6:**
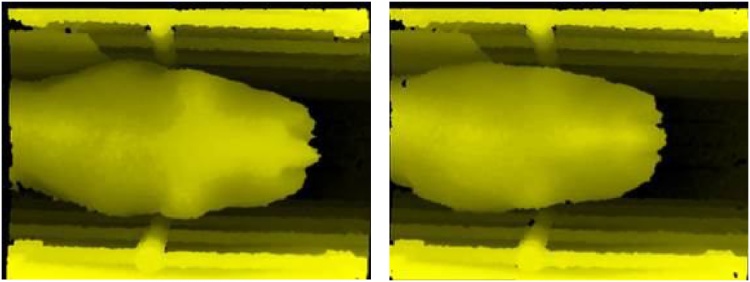
Manual BCS 2.75; system BCS 3.52 (left) and manual BCS 2.75; system BCS 3.47 (right).

**Fig. 7:**
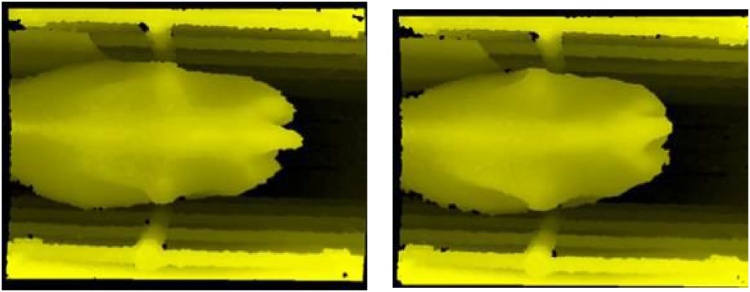
Two cows with a typical 2.75 BCS.

**Fig. 8:**
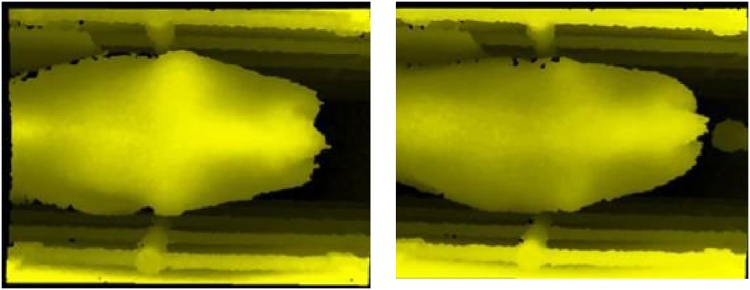
Two cows with a typical 3.5 BCS.

The presence of some discrepancy between the automated and manual scoring prompts the question of how reliable the manual scoring is and also the wider question regarding the utility of its use. Many prior studies have pointed to the difficulty in obtaining reliable ground truth data using manual observations and although various solutions have been proposed, this remains a weakness in any objective evaluation. Given that we have stored both 2D and 3D images, we have a useful opportunity here to revisit the raw manual scoring data for those cases where a discrepancy exists. Fig. 6 shows two cows manually scored as BCS 2.75 but estimated by the system as BCS ∼3.5, while Fig. 7, Fig. 8 show cows correctly estimated as BCS 2.75 and 3.5 respectively

It can be seen that the overestimated cows in Fig. 6 are far more like the 3.5 s depicted in Fig. 8. There is less definition along the spine and hooks than there is in the 2.75 s shown in Fig. 7, and no definition of the area above the rumen (from the hooks forward) as there is in the 2.75 s. It appears that the automated system has given a more accurate assessment than the human scorers. This observation was repeated across all the discrepancies. This would suggest that the automated system is working very well, providing a range of accurate BCS scores and that the manual scoring is inconsistent.

**Fig. 9:**
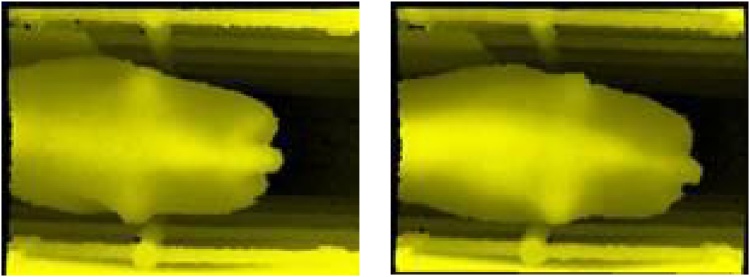
Manual BCS score – 3.5; system BSc score 3.22 (left) and manual BCS score 3; system BCS score 3.26 (right).

Another example concerns two cows that are scored differently by the observers but similarly by the system. Fig. 9 shows a clear example where the manual scoring suggests a half point reduction from 3.5 to 3 in BCS, while the system suggests no change. A half point change should be clearly visible, but is not.

#### 5.1.1 Repeatability – BCS

**Fig. 10:**
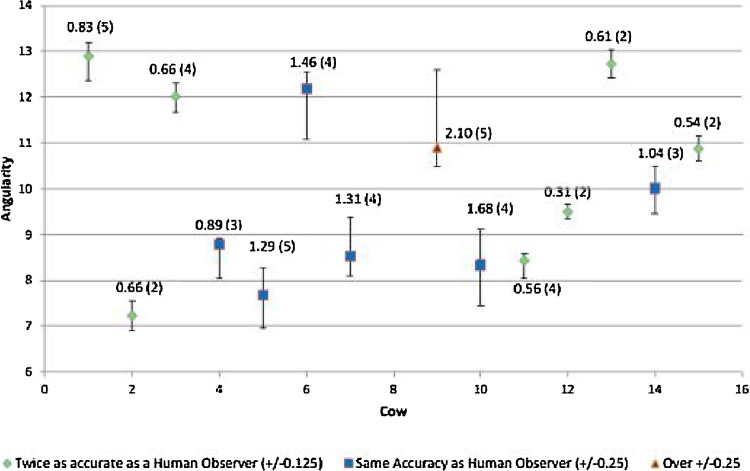
BCS for a group of 16 cows that passed through the system up to five times on the same day. Range of angularity is plotted for each cow with the number of repetitions given in parentheses. Increased angularity correlates with reduced body condition.

The test results in Fig. 10 show BCS for the group of 16 cows that passed through the system for up to five times on the same day. The figure shows an average range, ie the difference between the largest and smallest estimate across repeated viewing of the same cow, of 0.97.

Each quarter BCS can be represented by 0.89 angularity points. A range in angularity score of 1.88 therefore represents ±0.25. Experienced observers are unable to detect changes of ±0.25 over one time period [4], and hence the system presented here operates at or above this level.

### 5.2 Results – lameness using mobility assessment

**Fig. 11:**
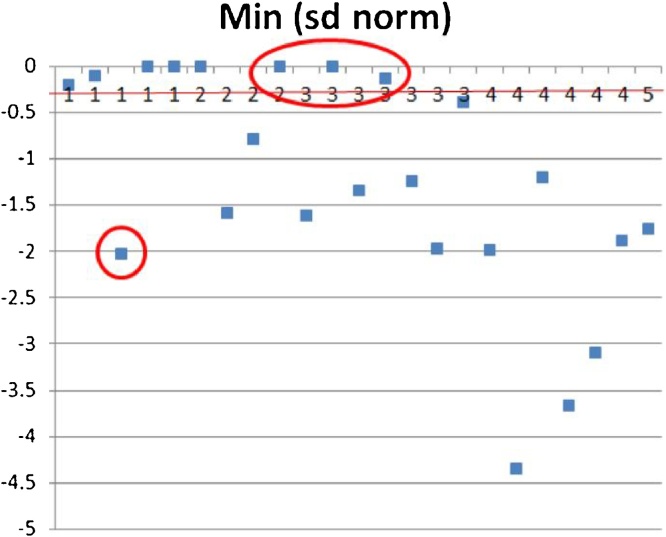
Mobility score (horizontal axis) plotted against normalised minimum B-term (Eq. (4)).

Fig. 11 shows mobility results for 23 cows, where each cow appears as a blue dot. All 23 cows were manually scored by trained observers, as shown by the score numbers 1–5 along the horizontal axis. Cows with a score of 3 or greater were considered as lame. The automated score result is shown on the vertical axis, where a negative value of −0.3 (indicated by the red horizontal threshold line) or greater indicates a lame animal. Of the 23 cows manually scored, 19 are correctly classified and 4 incorrectly classified. The 4 incorrectly classified cows are circled in red in the figure and comprise one false positive, where a healthy cow was classified as lame, and three false negatives, where lame cows were classed as healthy (one had a manual score of 2 and two had a manual score of 3).

### 5.3 Results – weight estimation

In terms of accuracy, the weights of 185 cows as measured by a set of calibrated scales (Tru-Test XR3000) were compared with estimates from the system. The error across all 185 cows is 6.1% (approx. ±18 kg for a 600 kg cow), with a regression score of 0.81 between estimated and actual.

In terms of repeatability, sixteen cows were weighed by the system. This number was chosen as a practical compromise in terms of the logistics of getting cows to pass beneath the system in short amount of time. After removing three outliers caused by cow bunching (readily detectable), the resulting weight estimates gave an average range of 78.18 kg, which is 11% of the mean cow weight (688 kg) or ±5%. 14 cows were also repeatedly weighed by the system up to 5 times within an hour, for which the average range across reading for a cow was 42.93 kg, which is 6.25% of the average weight or ±3.13%.

## 6 Discussion and conclusions

We have shown how for the first time all three cattle monitoring traits of body condition, lameness and weight, can be simultaneously obtained from a single data set captured using a single low-cost automated device. A new approach for 3D body condition assessment based on a rolling ball algorithm has been validated; achieving a repeatability of within ±0.25 BCS. While the manual use of a cow’s spinal curvature has long been used as an indicator for mobility scoring, the automatic extraction of the spine from accurate depth imagery has not been used before and was shown to achieve a classification accuracy of 83% (using a lameness threshold score of 3). Weight estimates were shown to be within ±5%. The results obtained under farm conditions in continuous operation were shown to be comparable or better than manual scoring of the herd. Shortcomings in manual scoring have been highlighted and the suggestion of using relative change in learned measurements over successive observations was proposed as an alternative approach.

## 7 Further work

Future work will explore localisation of limb lameness using further analysis of the 3D spine curve. Dynamic gait characteristics will also be added to the model, where an analysis of the depth map of the cow's back over successive frames allows for changes in the height of the animal in specific areas (e.g. over the limbs) to be conducted. A signal or beat of the gait can then be built up and any irregularities or asymmetries should be indicative of a limp.
